# Transcriptomic responses to predator kairomones in embryos of the aquatic snail *Radix balthica*

**DOI:** 10.1002/ece3.4574

**Published:** 2018-10-17

**Authors:** Oliver Tills, Manuela Truebano, Barbara Feldmeyer, Markus Pfenninger, Holly Morgenroth, Tilman Schell, Simon D. Rundle

**Affiliations:** ^1^ Marine Biology and Ecology Research Centre University of Plymouth, Drake Circus Plymouth UK; ^2^ Molecular Ecology Group, Institute for Ecology, Evolution and Diversity Goethe‐University Frankfurt am Main Germany; ^3^ Adaptation and Climate Senckenberg Biodiversity and Climate Research Centre Frankfurt am Main Germany; ^4^ Senckenberg Research Institute and Natural History Museum Frankfurt Frankfurt Germany; ^5^ LOWE‐TBG Centre for Translational Biodiversity Genomics Frankfurt Germany

**Keywords:** development and evolution, molluscs, phenotypic plasticity, transcriptomics

## Abstract

The ability of organisms to respond to predation threat by exhibiting induced defenses is well documented, but studies on the potential mechanistic basis for such responses are scarce. Here, we examine the transcriptomic response to predator kairomones of two functionally distinct developmental stages in embryos of the aquatic snail *Radix balthica*: E8—the stage at which a range‐finding trial indicated that kairomone‐induced accelerated growth and development first occurred; and E9—the stage at which embryos switched from ciliary‐ to crawling‐driven locomotion. We tested whether expression profiles were influenced by kairomones and whether this influence varied between stages. We also identified potential candidate genes for investigating mechanisms underpinning induced responses. There were 6,741 differentially expressed transcripts between developmental stages, compared to just five in response to predator kairomones. However, on examination of functional enrichment in the transcripts responding to predator kairomones and adopting a less stringent significance threshold, 206 transcripts were identified relating to muscle function, growth, and development, with this response being greater at the later E9 stage. Furthermore, these transcripts included putative annotations for genes identified as responding to predator kairomones in other taxa, including C1q, lectin, and actin domains. Globally, transcript expression appeared reduced in response to predator kairomones and we hypothesize that this might be a result of metabolic suppression, as has been reported in other taxa in response to predation threat.

## 1 INTRODUCTION

Predation pressure plays an important role in the ecology and evolution of prey species (Abrams, 2000). The production of defensive traits such as protective morphologies or avoidance behaviors often incurs significant costs that can lead to trade‐offs between traits (Koricheva, Nykänen, & Gianoli, 2004; Rundle & Brönmark, 2001). One way that prey can reduce the costs associated with defensive traits is by only expressing the defense when they are in an environment where the risk of predation is high. These so‐called induced defenses have been demonstrated in a wide variety of animal and plant taxa (Tollrian & Harvell, 1999). Their study has made a significant contribution to our understanding of topics related to phenotypic plasticity including associated costs (DeWitt, 1998; DeWitt, Sih, & Wilson, 1998), the relative importance of adaptive versus nonadaptive plasticity (Ghalambor et al, 2015), and the potential micro‐ (Scoville & Pfrender, 2010) and macro‐evolutionary importance (Van Buskirk, 2002) of plasticity.

Freshwater lakes and ponds are excellent models for studying the ecology and evolution of induced defenses. Levels of predation pressure can vary significantly among otherwise similar freshwater habitats within a region due to a combination of factors including glacial history, species introductions, dispersal dynamics, and habitat permanency (Stoks, McPeek, & Mitchell, 2003; van Buskirk, 2002). Furthermore, the isolated nature of many freshwater habitats contributes to their potential to facilitate local adaptation, including through processes such as genetic accommodation and genetic assimilation associated with phenotypic plasticity (Scoville & Pfrender, 2010). These two factors have led to a diverse range of examples of induced defenses in freshwater taxa including protective morphologies (tail depth in tadpoles; spine, and neck tooth production in *Daphnia* spp.; shell thickness and shape in gastropods), camouflage (melanism in *Daphnia* spp and gastropods), avoidance behaviors (diel vertical migration in *Daphnia* spp; avoidance behaviors in gastropods), and life history shifts (Lakowitz, Brönmark, & Nystrom, 2008; Sakwińska, 2002).

Despite the intense research effort in studying the evolutionary ecology of induced defenses in freshwater taxa, our knowledge of the mechanistic basis for this plasticity is less well understood. Studies of molecular‐level responses to predator kairomones in *Daphnia* spp. (Pauwels, Stoks, & Meester, 2005; Rozenberg et al, 2015; Schwarzenberger, Courts, & von elert E, 2009; Spanier et al, 2010), fish (Ghalambor et al, 2015; Mommer & Bell, 2014), and anurans (Cohen, Piacentino, & Warkentin, 2018; Cohen, Seid, & Warkentin, 2016) have provided an important first step in this regard. In *Daphnia* embryos, there is evidence that expression of induced defenses can occur during early development (Krueger & Dodson, 1981; Laforsch & Tollrian, 2004, 2004; Parejko, 1992; Weiss, Leimann, & Tollrian, 2015) and studies of molecular responses to predation threat have also identified functional responses that appear to be candidates for underpinning such morphological‐induced defenses (An, Do, Jung, Karagozlu, & Kim, 2018; Miyakawa et al, 2010).

Aquatic gastropods are a group that has been studied extensively for their induced defenses, with freshwater taxa in particular showing high levels of plasticity in response to predation threat (Bourdeau et al, 2015). The pond snail *Radix balthica* is natural prey of the fish *Tinca tinca* (O'Maoileidigh & Bracken, 1989) and responds phenotypically to predatory fish kairomones, including altered shell growth (Brönmark, Lakowitz, & Hollander, 2011) and modified behavior (Ahlgren & Brönmark, 2012) in adults. Furthermore, altered timing of developmental events in the presence of predatory fish kairomones during its embryonic development has also been described (Rundle, Smirthwaite, Colbert, & Spicer, 2011). The recent construction of developmental (Tills, Truebano, & Rundle, 2015) and adult (Feldmeyer, Wheat, Krezdorn, Rotter, & Pfenninger, 2011) transcriptomes for *R. balthica* and a draft genome for the congeneric *Radix auricularia* (Schell et al, 2017) provides a valuable opportunity to investigate induced defenses at both phenotypic and molecular levels.

Here, we investigate the ontogeny of molecular‐level responses of embryos to predatory fish kairomones. Our main aims were to assess: (a) whether transcript expression was influenced by embryonic exposure to predator kairomones; (b) if so, whether this effect varied between different points during ontogeny; and (c) to identify possible candidate genes for use as models for investigating mechanisms underpinning induced defense responses early in development. A recent meta‐analysis identified altered growth rate and increased shell production as two of the most common responses to predator kairomones in molluscs (Bourdeau et al, 2015). Consequently to identify when during embryonic development, altered growth and accelerated development occurs in response to predator kairomones in *R. balthica* we used range finding experiments and focused our analysis on two functionally distinct developmental stages during this period.

## 2 MATERIALS AND METHOD

### 2.1 Experimental treatments

Range‐finding experiments were first used to identify the developmental stages at which the phenotypic responses typically associated with predator kairomones (accelerated development and altered growth) occurred. Individual eggs, extracted from egg masses from a laboratory stock population, were maintained at 15°C in individual cell wells in 2 ml of either artificial pond water (ASTM, 1980) with 90 mg/L[Ca^2+^] or the same water containing predator kairomones. Predator kairomone water was created by maintaining an individual tench (*T. tinca*) from a stock population in 15 L of water (Dalesman, Rundle, Coleman, & Cotton, 2006). Embryos were examined and staged (using Cumin, 1972) every 24 hr under a dissecting microscope. The size of hatchlings at one and seven days after hatching was measured from digital images taken using a Nikon E4500 mounted on the microscope using UTHSCSA Image Tool for Windows.

Developmental rates were the same in predator and control treatments up to the E7 stage (mean time in both treatments: 5.2 days), but those in predator kairomones hatched significantly earlier than controls (mean hatching dates: control 11.3 ± 0.1; kairomones 10.4 ± 0.1 days; *F*_1,36_ = 23.51, *p* ≤ 0.001). There was no difference in shell length one day after hatching meaning that embryos in predator kairomones had accelerated growth and development between the E7 embryonic stage and hatching. Furthermore, this accelerated growth appears to have continued with hatchling snails at 7 days post‐hatch having significantly larger shells than controls (mean lengths: control 1.15 ± 0.04 mm; kairomones 1.34 ± 0.04 mm; *F*_1,30_ = 13.16, *p* ≤ 0.001).

**Figure 1:**
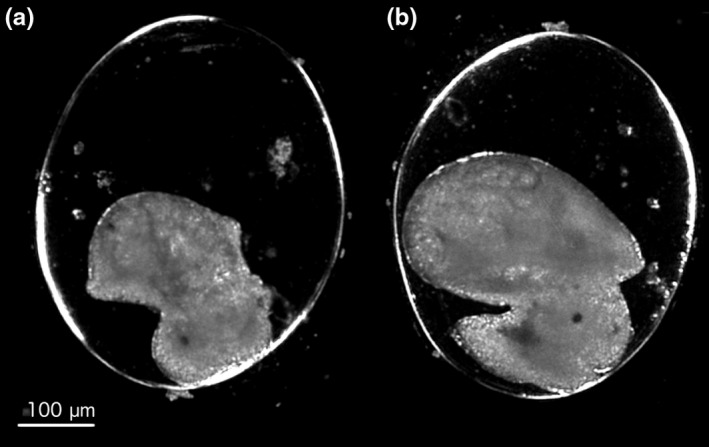
Micrograph images of *Radix balthica* embryos at (a) E8 and (b) E9, stages of development

Based on the range‐finding trials, we investigated transcriptomic responses at two developmental stages, E8 and E9 (Figure 1, Cumin, 1972), which fall on either side of the transition in the embryo from ciliary‐driven spinning behavior to crawling using the muscular foot. Heart contractions also begin at stage E8. At stage E9, the shell has grown over the entire visceral complex and the embryo has undergone torsion with the heart moving from the right to the left side of the body. These stages are separated by approximately 36 hr (at 20°C) in *R. balthica* embryos.

### 2.2 Sample preparation

Founding study animals were collected from a pond on Dartmoor, UK (50°27′54′′N, 4°02′12′′W), in January 2014 and were maintained in the laboratory at 15°C under a 12:12 light:dark regime (Tills et al, 2015). F_1_ *R. balthica* embryos were removed from this stock population and assigned to either a control (dechlorinated tap water, Ctrl) or predator kairomones treatment (Pred), which was prepared by keeping an individual tench, *T. tinca* (length 15–20 cm), for 1 hr in 5 L of dechlorinated tap water (Dalesman et al, 2006). Individual embryos were cultured in 0.6 ml of treatment water within a well in a 48‐well microtitre plate, positioned within an incubation chamber (*T* = 20 ± 0.1°C, Okolab, Naples, Italy). Daily water changes were performed via manual pipetting at which point embryos were staged. When embryos reached the developmental stage E8 (approx. 115 hr after 4‐cell division, Tills, Rundle, & Spicer, 2013a, 2013b) or E9 (approx. 130 hr after 4‐cell division), three replicate samples from each of the four treatments (Ctrl—E8, Ctrl—E9, Pred—E8, and Pred—E9) were constructed from pooled individual embryos (*n* = 150), fast‐frozen using liquid nitrogen, and subsequently stored at −80°C. Pooled samples were used with the aim of reducing biological variance between pools and providing a more representative treatment‐level molecular response (Diz, Truebano, & Skibinski, 2009) than was otherwise possible with the limited sample sizes in this study.

### 2.3 RNA extraction and bioinformatics

RNA was isolated and used to prepare libraries, which were then sequenced using both 250‐bp, paired‐end MiSeq (Illumina, San Diego, USA) and 50‐bp single‐read HiSeq 2000 (Illumina) (see Tills et al, 2015 for full details). MiSeq reads of each of the 12 samples (three for each of Ctrl—E8, Ctrl—E9, Pred—E8, and Pred—E9) were used to perform a de novo transcriptome assembly using Trinity (version r20140412), and this produced 116,197 transcripts (98,315 potential genes). These transcripts were filtered to remove those containing <1% expression, relative to the Trinity component to which it belongs and an FPKM of <1, resulting in 54,630 transcripts with a mean sequence length of 1,380 bases and an N50 of 2,474 (BioProject PRJEB9533). For more detailed information on RNA isolation and bioinformatics, see Tills et al (2015). Subsequent annotation using Trinotate (with an *e* ^−5^ threshold) saw seventy‐three percent of transcripts assigned putative annotations (Supporting Information Data S1). To obtain a functional overview of the transcriptome, all transcripts for which a Gene Ontology annotation was present had their GO categories collapsed and binned to the level of the generic GOSlim terms defined by the GO Consortium, using the goSlim function from the R package GSEABase (Subramanian et al. 2005, Supporting Information Data S2). See Supporting Information Figure S1 for mappings of annotated transcripts to different GOSlim categories.

HiSeq sequencing produced 121.19 M 50‐bp reads, and these were filtered to remove sequencing adapters and bases with a phred score <30 using Cutadapt (version 1.3, quality cutoff = 30; Martin 2011, Supporting Information Data S3). The resulting 121.15 M reads were subsequently mapped to the annotated reference transcriptome using the BWA‐MEM algorithm from the Burrow‐Wheeler Aligner software package (version 0.7.9a‐r786, Li, 2013), and differential expression analysis was performed.

Expression levels were quantified from the alignments using eXpress (v 1.5.1, https://bio.math.berkeley.edu/eXpress, Supporting Information Data S4). To identify patterns in the gene expression data and visually assess similarities between treatment groups, principal component analysis (PCA) of the 500 transcripts with the highest levels of variance across samples (Supporting Information Data S5) was performed using the package FactoMineR (Le, Josse, & Husson, 2008) in R. To describe which transcripts contributed to the separation of samples along each axis, expression levels were correlated with PCs 1 and 2 (dimdesc function, R package FactoMineR). To provide a functional overview, GO term annotations for these transcripts were collapsed and binned to the generic GOSlim terms (Supporting Information Data S6).

Differential expression was analyzed using the DESeq2 library (Love, Huber, & Anders, 2014) in R, with the default Benjamini–Hochberg adjusted *p*‐value cutoff of 0.1. The model tested was ~predator kairomones + developmental stage (predator kairomones treatments = control pond water, predator kairomones pond water; developmental stage = E8, E9). An interaction term was initially included in the analysis, but revealed no significantly differentially expressed (DE) genes and so was removed. The number of transcripts identified as differentially expressed in the predator kairomones treatment was low, and these were individually investigated and are detailed in the results. For GO term functional enrichment analysis, we adopted a relaxed *p*‐value (non‐adjusted *p* ≤ 0.01) to expand the number of transcripts, and the enrichment of these transcripts in their GO term annotations was compared against the functional profile of the entire transcriptome. This functional enrichment analysis was performed using the R package TopGO (version 2.20.0, Classic Test, Alexa & Rahnenfuhrer, 2010).

To assess the response of transcripts that might be underpinning phenotypic responses to predator kairomones related to increased growth rate, we filtered transcripts to those containing putative annotations for muscle, anatomical structure development, or lipid metabolic process. Transcripts associated with anatomical structure development and lipid metabolic process were identified by filtering transcripts to only those containing GO term annotations within these particular GOSlim categories (Supporting Information Data S2). Transcripts were considered to be associated with muscle if they contained at least one of the following muscle‐related GO terms: GO:0007525—somatic muscle development, GO:0016203—muscle attachment, GO:0003012—muscle system process, GO:0007014—actin ubiquitination, GO:0006936—muscle contraction, GO:0060537—muscle tissue development, GO:0061061—muscle structure development, GO:0006937—regulation of muscle contraction, GO:0055001—muscle cell development, GO:0048747—muscle fiber development, GO:0043292—contractile fiber, and GO:0000146—microfilament motor activity. To investigate molecular responses potentially underpinning altered shell development and growth, we used BlastN (*e* ≤ 10^−5^, Zhang, Schwartz, Wagner, & Miller, 2000) to identify transcripts in our assembly that showed similarity to the comprehensive list of 196 genes identified by Zhang et al (2012) as being involved in shell formation.

Candidate genes with treatment responses and putative annotations indicative of a functional response to predator kairomones were investigated further, that is, the presence of splice variants, by mapping to the draft genome assembly for *R. auricularia* (Schell et al, 2017) using HiSat2 v2.1.0 (Kim, Langmead, & Salzberg, 2015).

## 3 RESULTS

### 3.1 Global treatment overview

**Figure 2:**
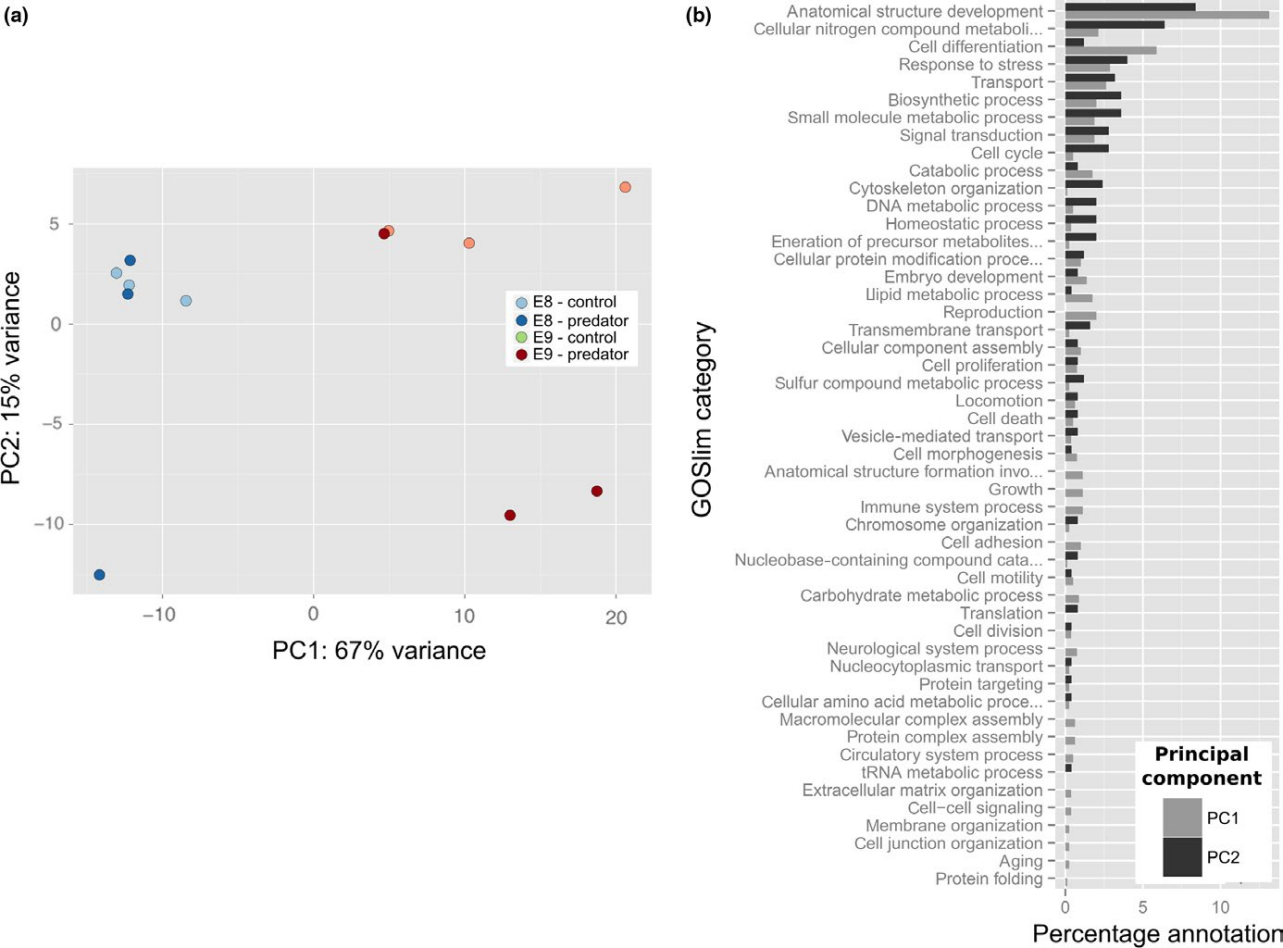
(a) Principal components 1 and 2 from a principal component analysis (PCA) performed on expression data from the 500 most variable transcripts within the *Radix balthica* transcriptome; (b) distribution of gene ontology (GO) annotations for transcripts identified by factor analysis as having a significant contribution to the separation of samples along PC1 or PC2 between the GO Consortium's Generic Biological Process GOSlim categories (Supporting Information Data S6). See Supporting Information Data S7 for vector plots visualizing the contribution of transcripts within different functional categories to sample separation along PCs 1 and 2

Principle component analysis (PCA) of the 500 transcripts with the highest levels of variance across samples revealed separation between treatments, but also high levels of variability between samples (Figure 2a). The first principle component (PC1) accounted for 70% of the variation in these 500 transcripts and broadly represented a separation of developmental stages, with the early stage E8 scoring lower than the later stage E9 on this axis. Transcripts correlated with PC1 had higher levels of annotation within the categories anatomical structure formation and cell differentiation (Figure 2b). PC2 accounted for 16% of the variation, and there was a general separation of predator and control treatments along this axis. Transcripts correlated with PC2 had higher levels of annotation within the categories anatomical structure formation and cellular nitrogen compound metabolic process (see Supporting Information Data S7 for correlation analyses for each axis, Supporting Information Data S8 for mapping of transcripts correlated with PC axes to different GOSlim terms and Supporting Information Figure S2 for biplots showing the contribution of these transcripts to sample separation).

### 3.2 Analysis of differentially expressed transcripts

**Figure 3:**
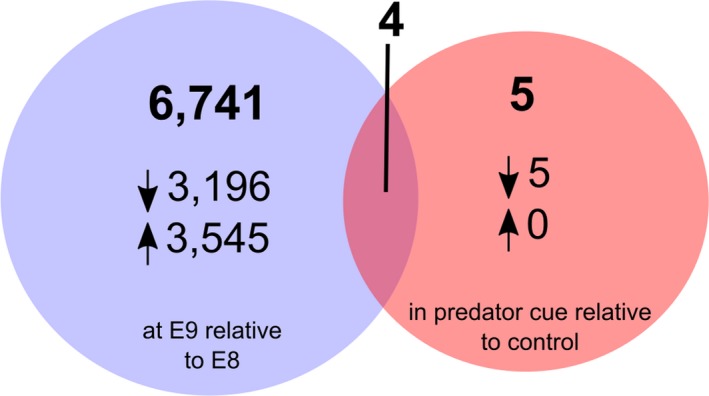
Venn diagram showing numbers of differentially expressed genes between developmental stages (heartbeat and crawling) and treatments (control and predator threat) (adjusted *p*‐value <0.1). The direction of the arrows indicates up‐ or down‐regulation

Comparison of differentially expressed (DE) transcripts between treatments revealed 6,741 DE transcripts that differed in their expression between the two developmental stages (Supporting Information Data S9) and five between the predator and control treatments (Benjamini–Hochberg *p*‐adj <0.1; Figure 3; Supporting Information Data S10). The five DE transcripts in response to predator kairomones were annotated and putatively identified as glutathione S‐transferase omega 1 (c32213_g2_i1), and genes containing EF hand (c33865_g1_i1, c33865_g2_i1), zinc‐finger/PB1 (c29648_g1_i1), or C1q (c16957_g1_i1) domains. Log fold change values and reaction norms (based on FPKM expression values) for these five transcripts showed that, in all cases, expression was down‐regulated at the E9 stage for those embryos developing in the presence of a predation threat (Figure 5). The response of these transcripts to predator kairomones was greatest in the E9 developmental stage with little response to predation threat evident in embryos at the, earlier, E8 stage. Of the five transcripts DE in response to predator kairomones, four were also differentially expressed between different developmental stages (transcripts c32113_g2_i1, c33865_g1_i1, c33865_g2_i1, and c16957_g1_i1).

**Figure 4:**
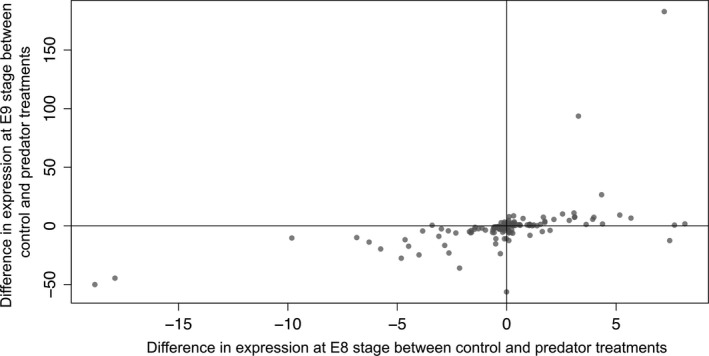
Differences in expression (DESeq2 rlog transformation) of transcripts between control and predator conditions at the E8 (*x*‐axis) and E9 (*y*‐axis) developmental stages. Positive and negative values indicate up‐ and down‐regulation, respectively

The five differentially expressed transcripts in the predator treatment were mapped to a draft genome for a congener *R. auricularia* to investigate whether there might be treatment‐specific splice variants. Transcripts c32113_g2_i1 and c29648_g1_i1 had the same exons covered in all four treatments. However, transcripts c33865_g1_i1/c33865_g2_i1 (EF hand) map to 3/2 exons respectively, and the additional exon of c33865_g1_i1 is expressed in only the E9 predator treatment. Transcript c16957_g1_i1 (C1q) (containing EF hand) was assembled as a single transcript; however, investigating stage‐specific exon expression, it becomes apparent that there are stage‐specific differences. We thus have evidence for stage‐specific splice variant expression for both EF hand‐containing genes (Supporting Information Data S11).

Within the predator treatment, only five transcripts were identified as being differentially expressed when using a Benjamini–Hochberg *p*‐adjusted threshold value of 0.1. Therefore, to identify gene functions that were potentially over‐represented in response to the predator treatment, we adopted a less conservative non‐adjusted *p*‐value threshold (≤0.01); this approach identified 206 such transcripts (Supporting Information Data S12). GO term functional enrichment applied to these transcripts revealed functional enrichment of GO terms indicative of positive regulation of skeletal muscle contraction, regulation of ryanodine‐sensitive calcium‐release channel activity, altered muscle development and function, positive regulation of feeding behavior, skeletal muscle tissue growth, neuromuscular process, positive regulation of skeletal muscle contraction, skeletal muscle contraction, skeletal muscle tissue growth, and actin filament fragmentation (Supporting Information Data S13). Furthermore, within these 206 transcripts, we also identified putative annotations for several genes that have previously been identified across taxa as responding to a predator threat, including genes coding for proteins with C1q, lectin, and actin domains (Pijanowska & Kloc, 2004; Rozenberg et al, 2015).

**Figure 5:**
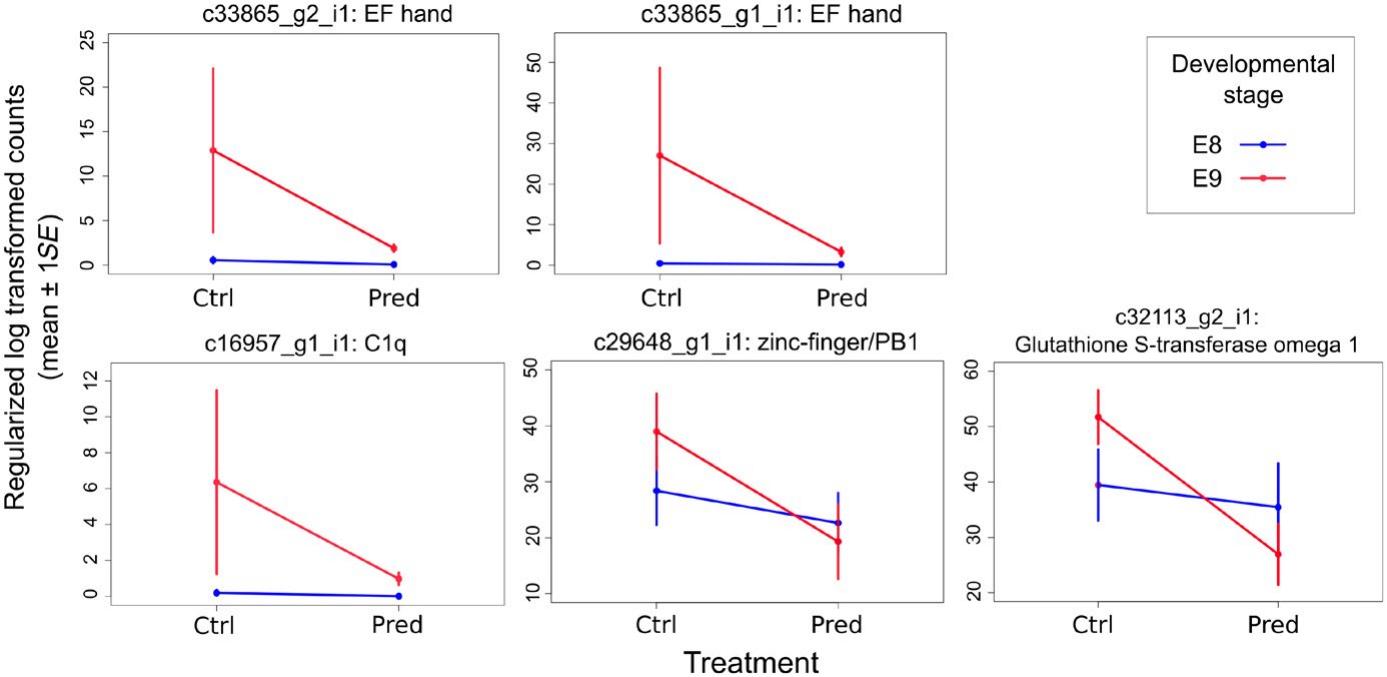
Effect plots for the five annotated transcripts that were differentially expressed between embryos in either control or predator conditions (mean rlog counts ± *SE*)

To address overall patterns of change in expression between control and predator conditions for the 206 DE transcripts (non‐adjusted *p*‐value of ≤0.01), we subtracted the mean expression value for each transcript in the control from the mean value in the predator treatment, for each developmental stage (Figure 4). At developmental stage E9, expression was lower in the predator treatment, as shown by more down‐regulated than up‐regulated transcripts (89 down‐regulated versus 55 up‐regulated at the E9 developmental stage; *χ* ^2 ^= 15.125, *p* = 0.0001. At developmental stage E8, there was no difference in expression level between predator treatments (78 down‐regulated versus 66 up‐regulated; *χ* ^2^ = 1.6806, *p* = 0.195). The largest proportion of transcripts down‐regulated in the predator‐exposed E9 stage appeared to reflect the reaction norm trends for annotated transcripts seen in Figure 5.

### 3.3 Transcriptional changes linked to shell formation

**Figure 6:**
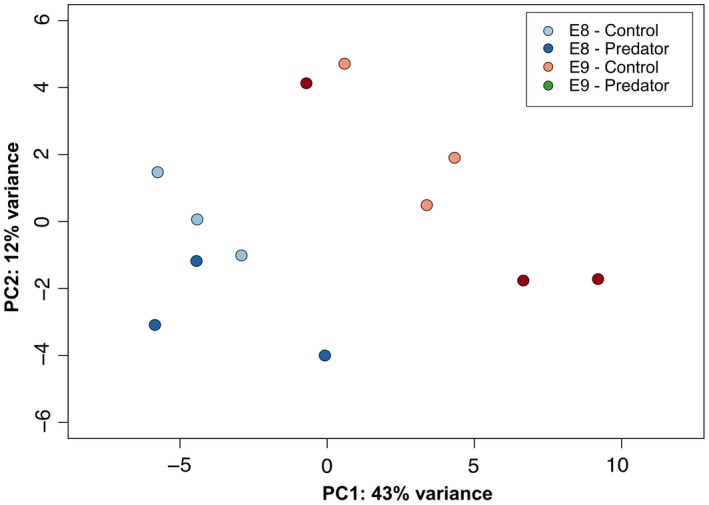
Principal component analysis of transcripts with a putative annotation suggestive of action in shell development

Zhang et al (2012) provide a comprehensive list of 196 genes involved in shell formation in molluscs. Here, we used this resource to identify transcripts from the *R. balthica* developmental transcriptome that could be involved in shell production and consequently whether their expression levels were affected by the presence of a predation threat. Using BlastN (*e* ≤ 10^−5^), we identified 74 transcripts with significant matches to the sequences from Zhang et al (2012) (Supporting Information Data S14). For several sequences, there were multiple transcripts with significant matches and therefore these were filtered by their *E*‐value to just the transcripts with the most significant match, resulting in 54 transcripts. Rlog expression values for these 54 transcripts were input to a PCA. The first principle component (PC1) from this analysis explained 43% of the variation in expression levels and gave a broad separation of samples for both developmental stages depending on exposure to predator kairomones (Figure 6).

### 3.4 Transcriptional changes linked to growth and accelerated metabolism

**Figure 7:**
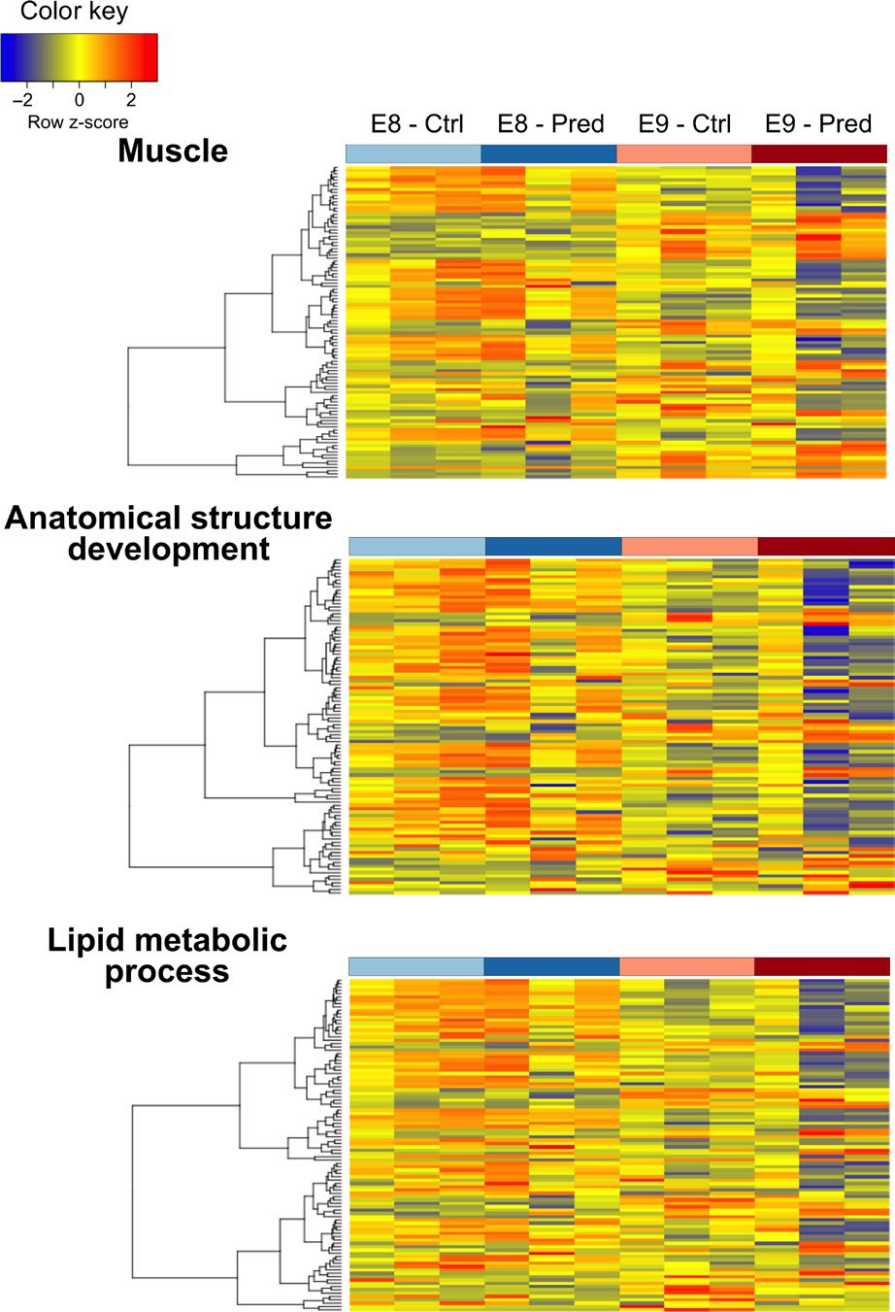
Heatmap (rlog expression levels) for the 100 transcripts with the greatest variance between samples containing the functional annotations: muscle (assorted GO terms—see Section 2), lipid metabolic processes (GOSlim category), and anatomical structure development (GOSlim category). See Supporting Information Data S15 for lists of transcripts and their annotations

Despite a focus in the current study on the effects of predator kairomones, there was some developmental stage‐specific clustering of transcripts with putative annotations indicative of growth and metabolism (muscle action, lipid metabolic processes, and anatomical structure development). Furthermore, there was also some differentiation evident at the latter E9 developmental stage between predator and non‐predator treatments, suggestive of these processes having some regulation in response to predation threat (Figure 7).

## 4 DISCUSSION

Our main aims in this study were to investigate whether exposure of embryos of the pond snail *R. balthica* to predator kairomones elicited differential expression of transcripts, and if so, whether these responses were developmental stage‐specific. We also aimed to identify candidate genes as models for investigating the mechanisms underpinning phenotypic responses to predator kairomones. We focused our analysis on functional categories associated with accelerated growth and increased shell growth (calcification), traits associated with responses to predators in molluscs, and confirmed by our range‐finding trial to occur in *R. balthica*. We identified broad‐scale transcriptome responses to predatory fish kairomones with reduced global expression at the latter E9 developmental stage. We also discovered developmental stage‐specific functional responses in the form of relevant functional categories, including muscle function and growth, and differential expression of genes identified as responding to predator threat in other taxa.

### 4.1 Functional overview

The greatest separation between samples, based on comparison of the 500 most variable transcripts, was evident between developmental stages. The transcripts contributing the most to this separation between samples had annotations indicative of cell differentiation and anatomical structure development, which is unsurprising given the increase in cell division and structural rudiment formation associated with later development (Cumin, 1972). However, there was also some separation of samples by the 500 most variable transcripts that appeared to reflect an influence of predator kairomones, with the transcripts that contributed most to this separation having functional annotations indicative of anatomical structure formation and cellular nitrogen compound metabolic process, suggesting some degree of resource re‐allocation in response to predators. Furthermore, there was an influence of predator kairomones on the expression of transcripts with putative annotations indicative of lipid metabolic processes, supporting the notion that these responses may contribute to the phenotypic responses of accelerated growth and development. Altered resource allocation has been inferred to occur in *Daphnia pulex* after long‐term exposure to predator kairomones (Rozenberg et al, 2015). In this case, a link was hypothesized between kairomone exposure and increased offspring size, through an increase in yolk production and a potential increase in expression of vitellogenin genes.

Our range‐finding studies with *R. balthica* demonstrated phenotypic effects of predatory fish kairomones in the form of accelerated growth and development during embryonic development from stage E8 up until hatching. Furthermore, at 7 days post‐hatch, snails cultured in the presence of predatory fish kairomones were larger. On exposure to predatory fish kairomones, *R. balthica* have been observed to exhibit modified frequency of particular behaviors, including crawling up out of the water or seeking refuge (Ahlgren & Brönmark, 2012), and some have suggested that predator‐induced morphological changes may be by‐products of these altered behaviors (Bourdeau & Johansson, 2012). Here, the demonstration of both altered growth rates from the embryonic stage E8 to 7 days post‐hatch and altered expression of genes relating to growth and development suggest a physiological underpinning to morphological effects of predator kairomones during early development in *R. balthica*. Furthermore, we identified the enrichment of functions associated with muscle development, growth, and function in response to predator kairomones. The E9 developmental stage marks a transition in the embryo from ciliary spinning locomotion to muscular crawling and the effect of predator kairomones appeared greatest at this stage, compared with the earlier E8 stage. Enrichment of these functions supports the hypothesis that differences in muscle development and function, perhaps related to predator avoidance, occur even during embryonic stages.

### 4.2 Analysis of differentially expressed transcripts upon predator exposure

Despite evidence of some global down‐regulation in response to predator kairomones, we identified only five differentially expressed transcripts within the predator treatment using the DESeq2 default significance threshold. The occurrence of a low number of transcripts being differentially expressed in response to predator kairomones has been shown previously in aquatic snails, where it contrasted with a high number of transcripts regulated in response to abiotic factors (Chu, Miller, Kaluziak, Trussell, & Vollmer, 2014). While the low number of DE transcripts could suggest a lack of universality of the response to environmental stressors when considering biotic factors, the possibility that chronic exposure to predator cues during development leads to acclimation cannot be excluded. Here, embryos were cultured in the continual presence of predator kairomones from four‐cell stage, and this could have resulted in transcript levels returning to control conditions after prolonged exposure. Analysis of gene expression following exposure to predatory kairomones at different temporal scales merits further investigation. Furthermore, it is also possible that responses to predation threat during development are subtle or highly variable, and cannot be fully captured with the sequencing depth and pooling approach applied in this study. Variation in developmental traits including developmental plasticity has previously been reported for *R. balthica* (Rundle et al, 2011; Tills et al., 2013a, 2013b, 2010; Tills, Spicer, & Rundle, 2010), and therefore, variation in the molecular response to predator kairomones is perhaps not surprising. Nonetheless, we acknowledge that this study has a low sample size (pools of 150 embryos, *N* = 3) and an associated limitation in statistical power and that this coupled with high levels of inter‐sample variability may have contributed to a limitation in our ability to detect more subtle treatment‐level responses.

Transcripts differentially expressed in response to predator kairomones were putatively identified as glutathione S‐transferase (GST) omega 1, and genes containing EF hand, zinc‐finger/PB1, or C1q domains. We propose that these transcripts would be interesting candidates for further analysis. GST is a member of a gene superfamily of multifunctional enzymes that catalyze the conjugation of glutathione (GSH) with numerous substrates. They facilitate the detoxification of endogenous and foreign chemicals and are up‐regulated in response to oxidative stress (Nebert & Vasilou, 2004). Omega class GSTs have been identified across taxa (Pearson, 2005) and catalyze the GSH‐dependent reduction in dehydroascorbate, monomethylarsonate, and protein disulfides, activities more characteristic of glutaredoxins. Previous studies have reported changes in antioxidant defenses under predation risk in invertebrates (Janssens & Stoks, 2013; Slos & Stoks, 2008), and these have been associated with antioxidant stress through an increase in oxygen consumption indicative of the fight‐or‐flight response (Janssens & Stoks, 2013). In embryos, changes in antioxidant defenses could be associated with an increase in respiration and metabolism, concordant with accelerated development, increased growth, and predator‐induced stress. In response to predator kairomones, adult *R. balthica* exhibit increased frequency of avoidance behaviors including emerging from the water and seeking refuges (Ahlgren & Brönmark, 2012). Furthermore, egg masses of *R. balthica* are typically laid submerged on either vegetation or substrate (pers. obs.), and hatchlings of *Lymnaea stagnalis*, a closely related species of *R. balthica*, have been shown to exhibit increased frequency of escape behaviors in response to predatory fish kairomones (Dalesman, Thomas, & Rundle, 2014). Consequently, an accelerated development and the differential expression of genes relating to detoxification may facilitate individuals exhibiting these escape behaviors earlier in their life history, thereby reducing predation threat. The relationship between predation risk and oxidative stress in snails is currently under investigation in our laboratory.

A transcript coding for a C1q domain‐containing protein was significantly down‐regulated in response to predator kairomones, with the most marked response being at the later E9 developmental stage. Furthermore, upon mapping reads to the genome of *R. auricularia*, we identified developmental stage‐specific splice variants for this gene (Supporting Information Data S11), indicating the usage of different proteins in the two stages. The C1q domain‐containing proteins are a family of proteins characterized by a globular C1q (gC1q) domain in their C‐terminus (Carland & Gerwick, 2010). They are involved with several processes in vertebrates and are associated with the innate immune system response in molluscs (Bulat et al, 2016; Castillo, Salazar, & Joffe, 2015; Gestal, Pallavicini, Venier, Novoa, & Figueras, 2010; Zhang et al., 2008). While their differential expression here could indicate stress‐induced immunosuppression due to lowered allocation of resources to immune function in response to predators, we observed no significant effects of predator kairomones in other genes associated with innate immunity. Interestingly, proteins of unknown molecular function, but also containing C1q domains, were over‐represented among those significantly down‐regulated in response to predators in *D. pulex* (Rozenberg et al, 2015).

### 4.3 Transcriptional changes linked to shell formation

Here, we identified the response of transcripts to predator kairomones with putative annotations indicative of shell formation (Zhang et al, 2012). Included in these annotations were *Calmodulin* (Peng, Liu, Wang, & Hong, 2017), several genes encoding for nacrein‐like proteins (Fang et al, 2011), and also the transcription factor *engrailed* (Moshel, Levine, & Collier, 1998). A response of transcripts underpinning processes involved in shell formation supports our observation at the phenotypic level, during range‐finding studies that shell development was accelerated during the embryonic period and beyond, up to 7 days post‐hatch. Predator‐induced morphological plasticity has been demonstrated in a range of taxa, and plasticity in shell morphology has been described in a wide range of molluscs species (Bourdeau, 2009; DeWitt, 1998; Hoverman, Auld, & Relyea, 2005; Zhang et al, 2012) and has included demonstration of the ability of mollusc to tailor their shell morphology to minimize the risk posed by multiple predators (Bourdeau, 2009; Hoverman & Relyea, 2007). The shell morphology of *R. balthica* has high levels of plasticity and consequently cannot be used to reliably identify the species, particularly as population‐specific shell forms are common (Pfenninger, Salinger, Haun, & Feldmeyer, 2011; Schniebs, Glöer, Vinarski, & Hundsdoerfer, 2011).

### 4.4 Transcriptional changes linked to growth and accelerated metabolism

Our range‐finding experiment identified accelerated growth in response to predator kairomones, from the embryonic developmental stage E8. An accelerated growth rate continued into post‐embryonic development and was evident at 7 days after hatching. Interestingly, at the molecular level, we identified broad differences in the expression levels of transcripts containing GO annotations within the broader GOSlim categories anatomical structure development and lipid metabolic processes. Accelerated development in response to predation risk has been demonstrated in embryonic stages of three‐spined stickleback alongside the differential expression of genes involved in functions indicative of accelerated development (Mommer & Bell, 2014).

## 5 CONCLUSION

In this study, using embryonic stages of *R. balthica*, we have identified the differential expression of transcripts indicative of functions underpinning phenotypic responses to predator kairomones. We find the greatest difference in expression to be between developmental stages separated by just 36 h, but there was also evidence of developmental stage‐specific responses with the later E9 stage exhibiting a clearer separation in our predator kairomones treatment. We identify a number of candidate genes for future research into the mechanisms underpinning responses to predation threat. Our study indicates that changes in gene expression in a small number of genes are sufficient to mediate distinct predator‐induced phenotypic differences observed in *R. balthica*.

## AUTHORS’ CONTRIBUTIONS

OT, SDR, MT, and HM contributed to study conception and design. OT, SDR, MT, and HM performed the laboratory trials. MT performed RNA extraction. MT, OT, BF, TS, and MP contributed to computational analysis. All authors contributed to preparing the manuscript.

## DATA ACCESSIBILITY

Transcriptome assembly: Accessible in the Zenodo data repository—https://doi.org/10.5281/zenodo.1407973.

RNA sequences: NCBI BioProject PRJEB9533.
