# Supplementary figures and images for: Transcriptomic responses to predator kairomones in embryos of the aquatic snail Radix balthica
Source: Ecol Evol. 2018 Oct 17;8(22):11071–82. doi: 10.1002/ece3.4574 (PMC6262742; doi:10.1002/ece3.4574)

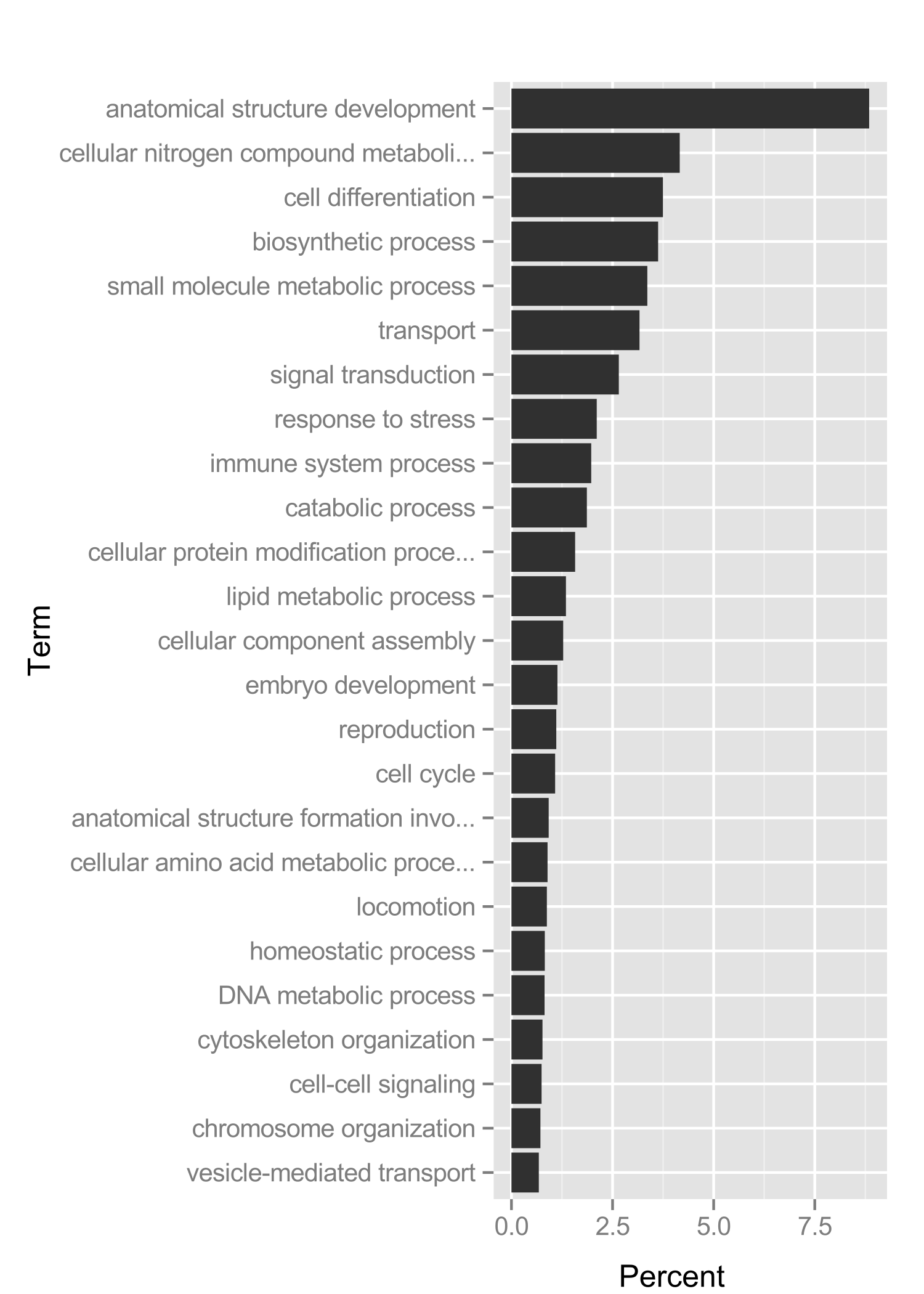

Supplement: Supplementary file 1 [file ECE3-8-11071-s001.docx]
