# Supplementary material for: Transcriptomic responses to predator kairomones in embryos of the aquatic snail Radix balthica
Source: Ecol Evol. 2018 Oct 17;8(22):11071–82. doi: 10.1002/ece3.4574 (PMC6262742; doi:10.1002/ece3.4574)
Supplement: Supplementary file 2 [file ECE3-8-11071-s002.pdf]

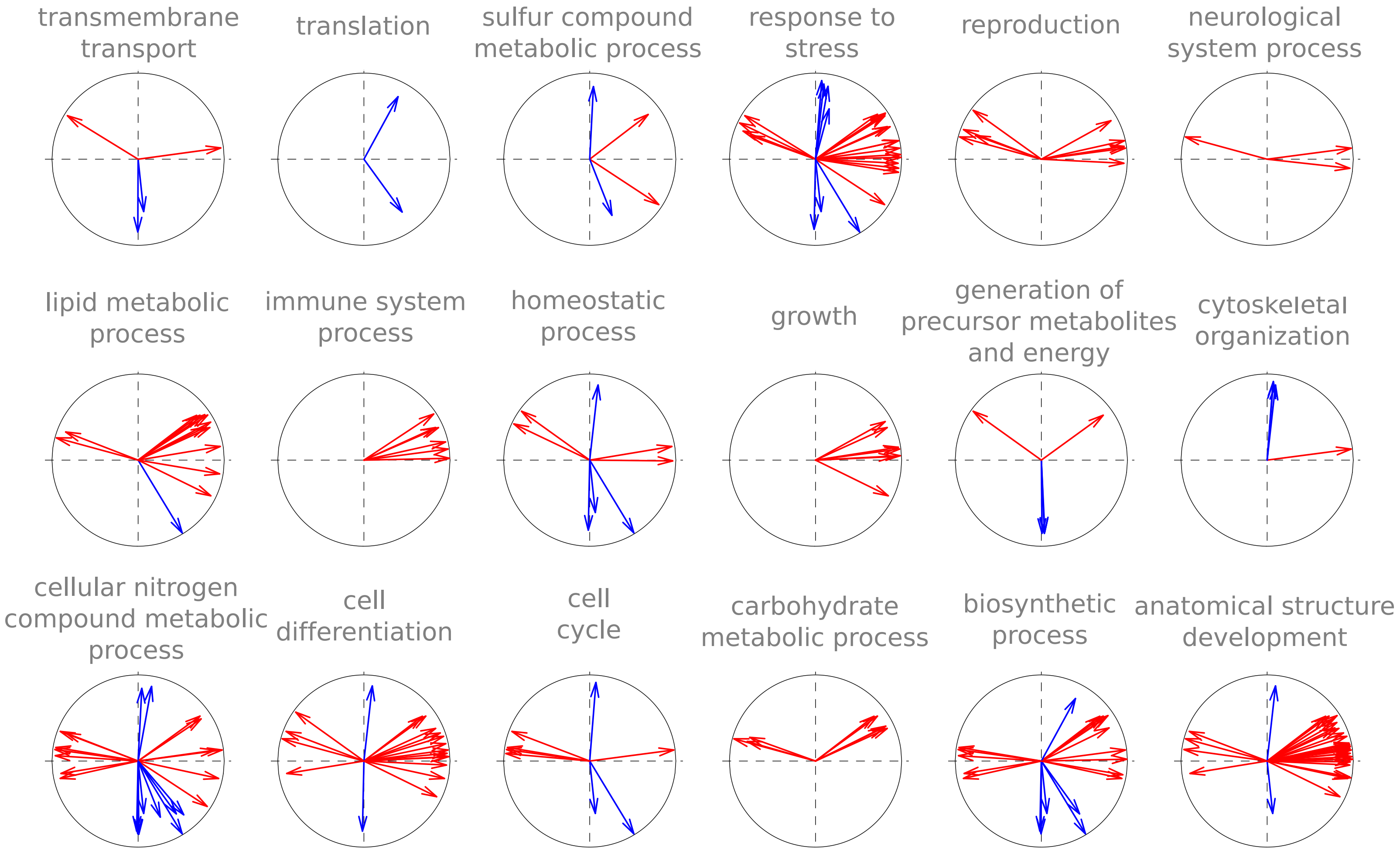
transmembrane transport
translation
sulfur compound metabolic process
response to stress
reproduction
neurological system process
lipid metabolic process
immune system process
homeostatic process
growth
generation of precursor metabolites and energy
cytoskeletal organization
cellular nitrogen compound metabolic process
cell differentiation
cell cycle
carbohydrate metabolic process
biosynthetic process
anatomical structure development
